# Brown Adipose Tissue Quantification in Human Neonates Using Water-Fat Separated MRI

**DOI:** 10.1371/journal.pone.0077907

**Published:** 2013-10-30

**Authors:** Jerod M. Rasmussen, Sonja Entringer, Annie Nguyen, Theo G. M. van Erp, Ana Guijarro, Fariba Oveisi, James M. Swanson, Daniele Piomelli, Pathik D. Wadhwa, Claudia Buss, Steven G. Potkin

**Affiliations:** 1 Department of Psychiatry and Human Behavior, University of California Irvine, Irvine, California, United States of America; 2 Department of Pediatrics, University of California Irvine, Irvine, California, United States of America; 3 Department of Anatomy and Neurobiology, University of California Irvine, Irvine, California, United States of America; 4 Department of Obstetrics & Gynecology, University of California Irvine, Irvine, California, United States of America; 5 Department of Epidemiology, University of California Irvine, Irvine, California, United States of America; 6 Drug Discovery and Development, Instituto Italiano de Tecnologia Italian, Genoa, Italy; 7 Department of Medical Psychology, Charité, Berlin, Germany; The University of Chicago, United States of America

## Abstract

There is a major resurgence of interest in brown adipose tissue (BAT) biology, particularly regarding its determinants and consequences in newborns and infants. Reliable methods for non-invasive BAT measurement in human infants have yet to be demonstrated. The current study first validates methods for quantitative BAT imaging of rodents *post mortem* followed by BAT excision and re-imaging of excised tissues. Identical methods are then employed in a cohort of *in vivo* infants to establish the reliability of these measures and provide normative statistics for BAT depot volume and fat fraction. Using multi-echo water-fat MRI, fat- and water-based images of rodents and neonates were acquired and ratios of fat to the combined signal from fat and water (fat signal fraction) were calculated. Neonatal scans (n = 22) were acquired during natural sleep to quantify BAT and WAT deposits for depot volume and fat fraction. Acquisition repeatability was assessed based on multiple scans from the same neonate. Intra- and inter-rater measures of reliability in regional BAT depot volume and fat fraction quantification were determined based on multiple segmentations by two raters. Rodent BAT was characterized as having significantly higher water content than WAT in both *in situ* as well as *ex vivo* imaging assessments. Human neonate deposits indicative of bilateral BAT in spinal, supraclavicular and axillary regions were observed. Pairwise, WAT fat fraction was significantly greater than BAT fat fraction throughout the sample (Δ_WAT-BAT_ = 38%, *p*<10^−4^). Repeated scans demonstrated a high voxelwise correlation for fat fraction (R_all_ = 0.99). BAT depot volume and fat fraction measurements showed high intra-rater (ICC_BAT,VOL_ = 0.93, ICC_BAT,FF_ = 0.93) and inter-rater reliability (ICC_BAT,VOL_ = 0.86, ICC_BAT,FF_ = 0.93). This study demonstrates the reliability of using multi-echo water-fat MRI in human neonates for quantification throughout the torso of BAT depot volume and fat fraction measurements.

## Introduction

There is a major resurgence of interest in brown adipose tissue (BAT) biology consequent to relatively recent discoveries that BAT persists into adulthood and appears to play a protective role against obesity/adiposity risk and metabolic dysfunction [1]–[3]. The nature of BAT as a specialized heat-producing and energy- expending tissue and its role in neonatal thermogenesis has long been established. However, very little is known about BAT characteristics in early life such as amount, activity, change over time, as well as its immediate and long-term implications for obesity/adiposity and metabolic function. These questions require the development of reliable and valid non-invasive methods to perform quantitative BAT imaging in newborns, infants and children.

In adults, the predominant imaging modality for BAT has been positron emission coupled with computed tomography (PET/CT) due to it’s ability to image active BAT metabolism alongside composition using Hounsefield unit attenuation. However, because PET/CT utilizes an ionizing source of radiation, it is not suitable for research use in pediatric populations. Despite it’s ability to only morphologically characterize BAT, magnetic resonance imaging (MRI) has been identified as the “next major advancement” in imaging BAT, particularly in pediatrics [4]. In contrast to the uni-locular adipocyte composition of white adipose tissue (WAT), BAT has multi-locular adipocytes, is mitochondria-rich, and capillary-dense. Consequently, the ratio of water to fat is greater in BAT relative to WAT, making it ideally suited for multi-echo water-fat MRI techniques.

Fat protons resonate at a frequency 3.5 parts per million higher than water, enabling BAT to be spectroscopically [5] differentiated from WAT. Multi-echo water-fat MRI takes advantage of the frequency difference, or chemical shift, by observing the contributions of differing phase on the overall signal [6]–[8]. Multi-echo water-fat MRI has localized rodent interscapular BAT (iBAT) [9], [10] and demonstrated increased water content between *in vivo* and *post mortem* states [11]. Rodent BAT volume quantification, derived from T2-weighted imaging with and without fat suppression, has further validated associations between iBAT and total weight [12] and between fat fraction and total weight [13]. Collectively, these studies demonstrate that rodent BAT has an MR signature unique from WAT.

Literature on quantitative BAT imaging in infants is very limited. Thermographic techniques have been used to quantify BAT heat generation in infants [14]. However, while thermography is reflective of BAT function, it does not provide quantitative information about BAT deposition. Three of the earliest MRI applications to measuring BAT are clinical case reports. The first report [15] in a single subject using T2-weighted imaging found abnormally large and clinically relevant BAT deposits extending from the lower neck to axillary regions. The second report in a *post mortem* infant used multi-echo water-fat MRI [16] and validated multi-echo water-fat MRI-based identification of BAT with CT and dissection. A third report identified an interscapular BAT depot in eight *post mortem* infants using MRI and verified the depots as brown fat using histological and biochemical analysis [17]. More recently, two studies have demonstrated the feasibility of using MRI to detect *in vivo* BAT deposits. These reports [18], [19], in 2 and 12 infants respectively, found lower *in vivo* BAT fat fractions relative to WAT and discrete deposits containing a high concentration of both adipocytes and water (consistent with BAT) in the supraclavicular fossa.

The goal of the present study was to assess the reliability of *in vivo* multi-echo water-fat MRI for quantification of BAT depot volume and fat fraction in human infants and characterize BAT deposition throughout the neonate torso. The protocol was first validated by *in situ* and *ex vivo* fat fraction comparison in rodents. Identical methods were then applied to neonates, *in vivo*, establishing normative measures of BAT depot volume and fat fraction throughout the torso. Two aspects of reliability were assessed and are reported here: test-retest reliability of the MRI data acquisition (in the same neonate), and intra/inter-rater reliability of BAT depot volume and fat fraction quantification (across subjects).

## Methods

### Rodent Imaging of BAT

This study was executed according to the recommendations found in the Guide for the Care and Use of Laboratory Animals of the National Institutes of Health. The protocol was approved by the Institutional Animal Care and Use Committee of the University of California at Irvine. Six two-month old Sprague-Dawley rats (250–275 g) were imaged *post mortem* simultaneously in a sealed and decontaminated container. Deposits of iBAT were then excised from the interscapular region along with WAT deposits from the perirenal region. The excised tissue was placed in glass tubes and immediately imaged. All imaging occurred within a three-hour period of animal sacrifice to minimize *post mortem* effects on the tissue. MRI was performed on a Siemens 3 T Tim Trio system (VB17 software) using a 12-channel head coil. Scans were conducted using a vendor-supplied chemical-shift two-point 3D gradient echo [20] Dixon method (TR = 7.47 ms, TE1/TE2 = 2.45/3.675 ms, NA = 16, BW/pixel = 977 Hz, FA = 10, Matrix = 512×320×160, 0.97×0.97×1 mm, Scan Time = 3 min 2 s).

### Neonatal Imaging

Infant imaging was approved by the Institutional Review Board of the University of California at Irvine, and all parents provided informed, written consent. After feeding and soothing to the point of sleep, neonates were placed in a CIVCO beaded pillow (www.civco.com), covering body and head, that becomes rigid under vacuum providing a comforting swaddle, motion prevention and hearing protection when used in conjunction with standard foam earplugs. A pediatric specialist throughout the duration of scans observed neonates, monitoring for heart rate and oxygen saturation via a pulse oximeter attached to the foot. Scans were aborted in all cases of wakefulness within the neonate. The entire protocol included T1-weighted, T2-weighted, diffusion tensor and functional imaging of the brain. Of the 25 subjects who were initially imaged, 3 were excluded prior to analysis due to fat/water swapping arising from the ambiguity of phase greater than 2π [21], [22] in the regions that contain BAT depots identified in this work (supraclavicular, axillary, spine), resulting in a final sample of 22 subjects (9 males and 13 females). All infants were from healthy pregnancies with no known obstetric, birth or current health complications. In terms of race/ethnicity approximately one half of the study sample was Non-Hispanic White (N = 13), whereas the other infants were either Hispanic White (N = 6) or Hispanic of other race (N = 3). The mean infant age at assessment was 23.6±11.7 (±SD) days and ranged from 11 to 56 days. Subjects were imaged during natural sleep using a combination of a 12-channel head receive coil and a posterior neck coil. An anterior neck coil was added to increase the signal to noise ratio when infant size could accommodate it (N = 17). The field of view was defined as just superior to the lower jaw down to the lower abdomen, with a fixed size. Imaging parameters were matched to the rodent imaging parameters, albeit with a single average resulting in a shorter scan time to minimize *in vivo* motion artifact.

### Image Processing and Segmentation

**Figure 1:**
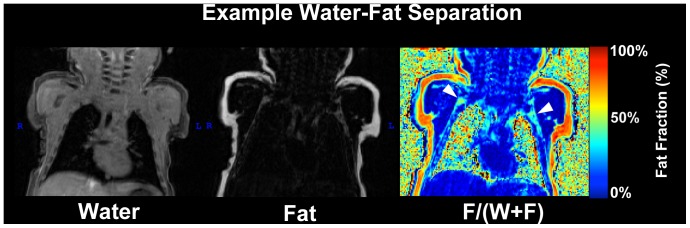
Example Water, Fat and Fat Signal Fraction Images. Example images of water (left) and fat (middle) separation based on the opposed-phase imaging are shown. Fat signal fraction (right) is defined as the ratio of fat to the combined signal from fat and water. Moderate fat fraction values indicate BAT depots in the supraclavicular/axillary region (white arrows).

**Figure 2:**
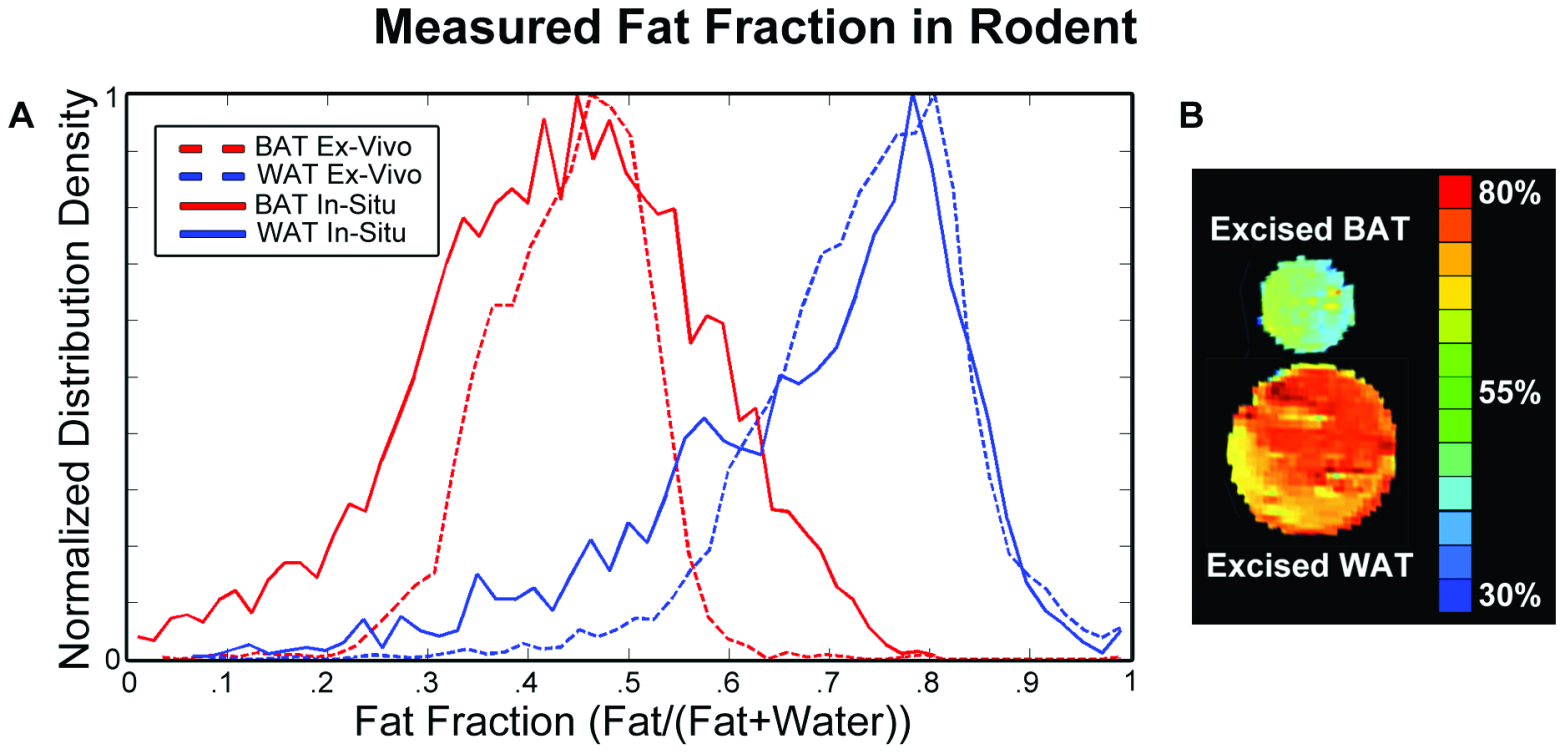
Fat Fraction in Rodent. Measured fat fraction in rodent demonstrates differentiation in fat fraction between white and brown adipose tissues. (A) *In situ* and *ex vivo* samples of BAT (brown adipose tissue) and WAT (white adipose tissue). (B) Single slice fat fraction map of *ex vivo* tissues in glass vials.

Voxelwise fat fraction (FF) maps were created from water and fat separated volumes (Figure 1) derived directly from the Siemens operating system, a feature readily available on most clinical platforms. The ratio of fat to the combined signal intensity from both fat and water used in this work is a fat signal fraction based on a two-point excitation incapable of accounting for the multiple peak resonances of fat and indirect dipole-dipole coupling. Hand drawn ROIs were used to extract FF from excised tissue samples and *post mortem* BAT regions (Figure 2). Thresholds for classifying BAT were determined based on a receiver operator characteristic analysis of the *ex vivo* samples. At an upper threshold of 60% FF, BAT was correctly classified 99% of the time with a WAT false positive of less than 10%. At a lower threshold of 20% FF, BAT was correctly classified 99.9% of the time.

**Figure 3:**
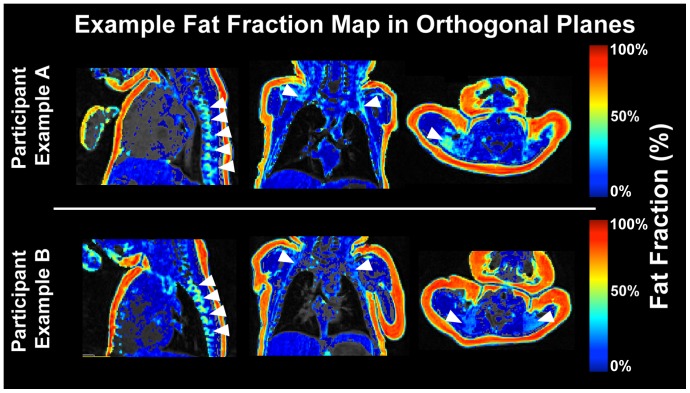
Fat Fraction In Three Planes. Three-plane view of BAT (brown adipose tissue) in a participant with relatively large (bottom row) and small (top row) deposits. Arrows in the axial and coronal views indicate supraclavicular/axillary BAT. Arrows in the sagittal view indicate bilateral spinal BAT deposits. Underlay volume is the in-phase echo.

Neonate BAT masks were created for visualization based on the following voxel attributes: 1) total signal greater than two standard deviations (SD) above the entire image mean (including background noise), 2) fat signal greater than one SD below the entire image mean, and 3) FF greater than 20% with no upper bound. Fat fraction values were overlaid on in-phase volumes to highlight individual differences in BAT depot volume, distribution and composition between subjects (Figure 3).

**Figure 4:**
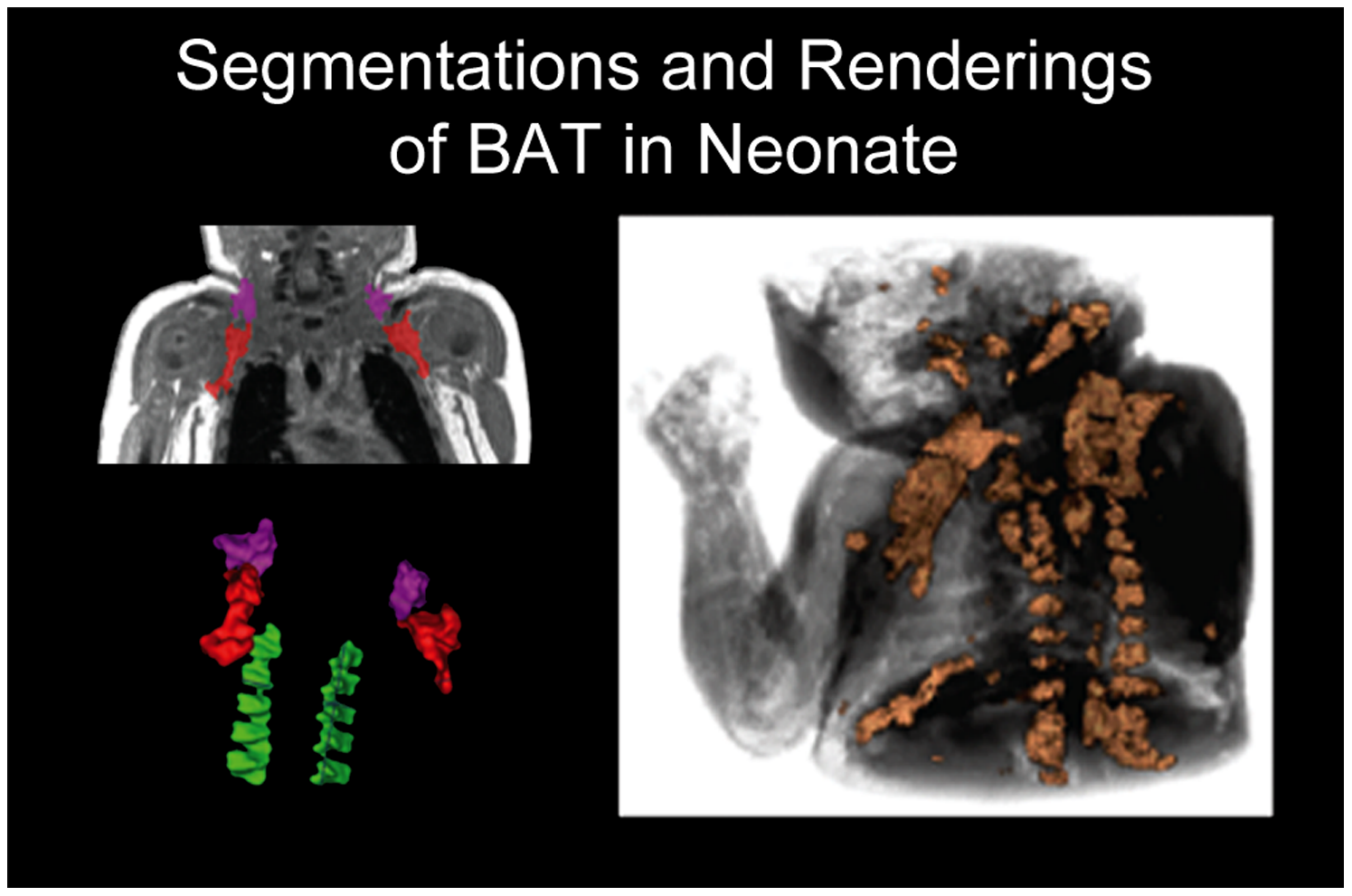
Neonatal Segmentations. BAT (brown adipose tissue) segmentations in the neonate. From left to right: sagittal view of semi-automatic segmentations of spine T1–T5 (green), coronal view of supraclavicular (magenta) and axillary (red) ROIs, the 3-dimensional render of ROIs used in this manuscript, a 3-dimensional render of all voxels that fit the BAT criteria used in this manuscript for visualization (rust color).

BAT and WAT deposits were segmented (Figure 4) and quantified for depot volume and fat fraction in all subjects using a semi-automated intensity-based process [23]. BAT was segmented in ITK-SNAP (www.itksnap.org) by applying a threshold filter limiting FF values between 20% and 60%. Seed points for active contour segmentation were manually placed in deposits close to lateral processes along the spine and in supraclavicular, and axillary regions. Seed bubbles of 3 mm were used to roughly cover these areas while avoiding any subcutaneous WAT partial volume regions. Supraclavicular ROIs were limited to four seed points per side, axillary to four seeds per side, and one seed per side on each vertebrae, ensuring consistent definition. Vertebral ROIs were limited to the first five thoracic vertebrae (T1–T5) in order to maintain well-defined and consistent boundaries for definition between raters. The active contour evolution was iterated until the contours were visually covered by label, between 40 and 60 iterations. Iterations were constrained to 50 in the more heterogeneous supraclavicular and axillary regions to avoid bleeding into neighboring voxels. WAT was segmented using the fat-only image and thresholding for values above the lowest quartile. A single seed of 5 mm was placed in the subcutaneous nuchal area of the neck to avoid regions contaminated by fat and water signal swap. The active contour evolution was iterated 100 times. Mean FF and total depot volume were computed for each of the three segmented BAT regions and one nuchal WAT region. An additional ROI mask containing the union of supraclavicular and axillary regions was also assessed to account for the occasionally ambiguous border between the supraclavicular and axillary regions.

## Reliability

**Figure 5:**
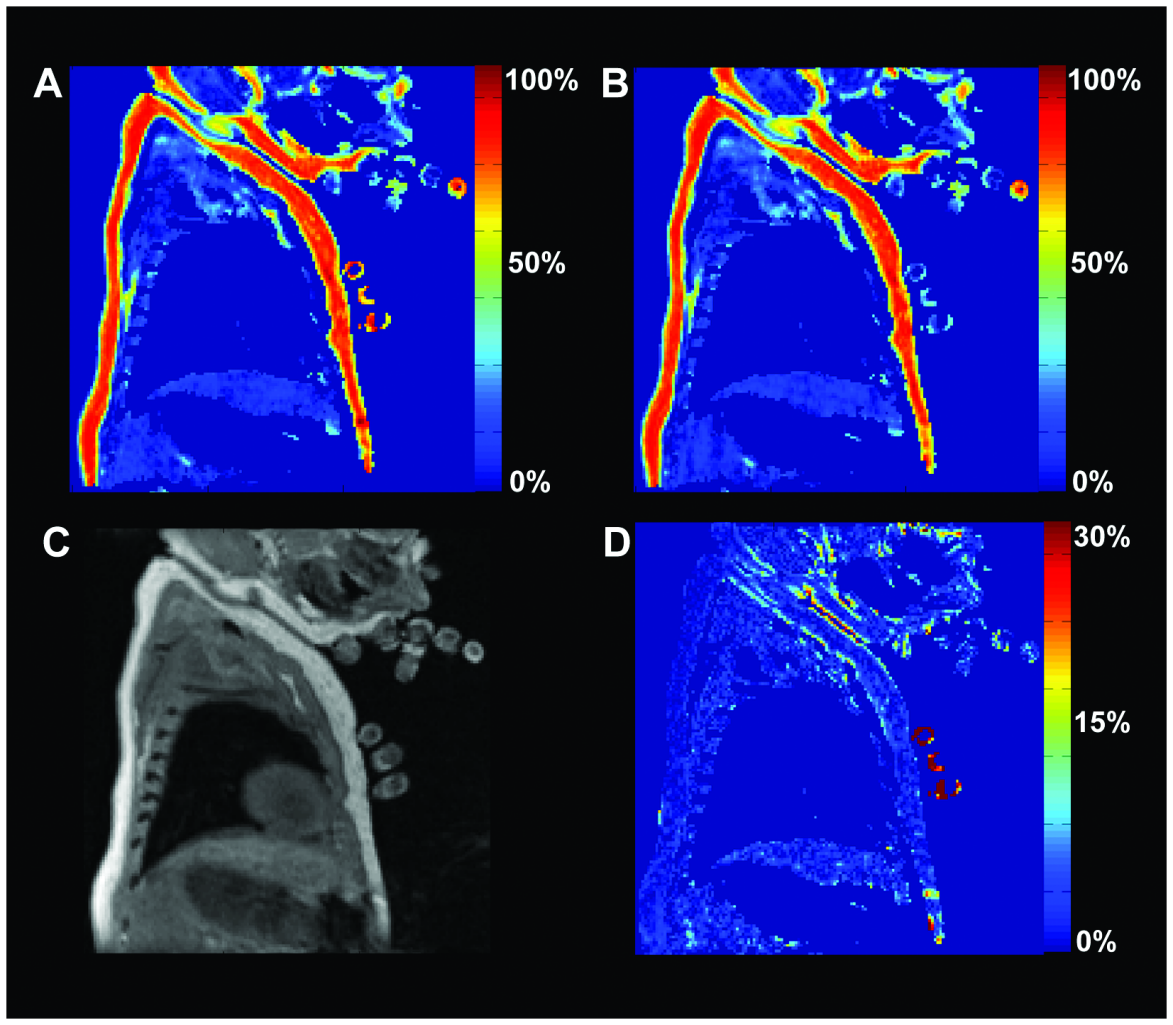
Acquisition Repeatability. Sagittal view of scan/rescan comparison of Dixon based fat fraction maps in a single neonate. (A) First scan followed by (B) re-shimming and an identical repeat scan, hotter color denotes a higher fat fraction. (C) In-phase image depicting underlying anatomy. (D) Difference map in calculated fat fraction, note highest intensities are found at edges due to mis-registration between scans and an inhomogeneity artifact present in the fingers anterior to the chest.

**Figure 6:**
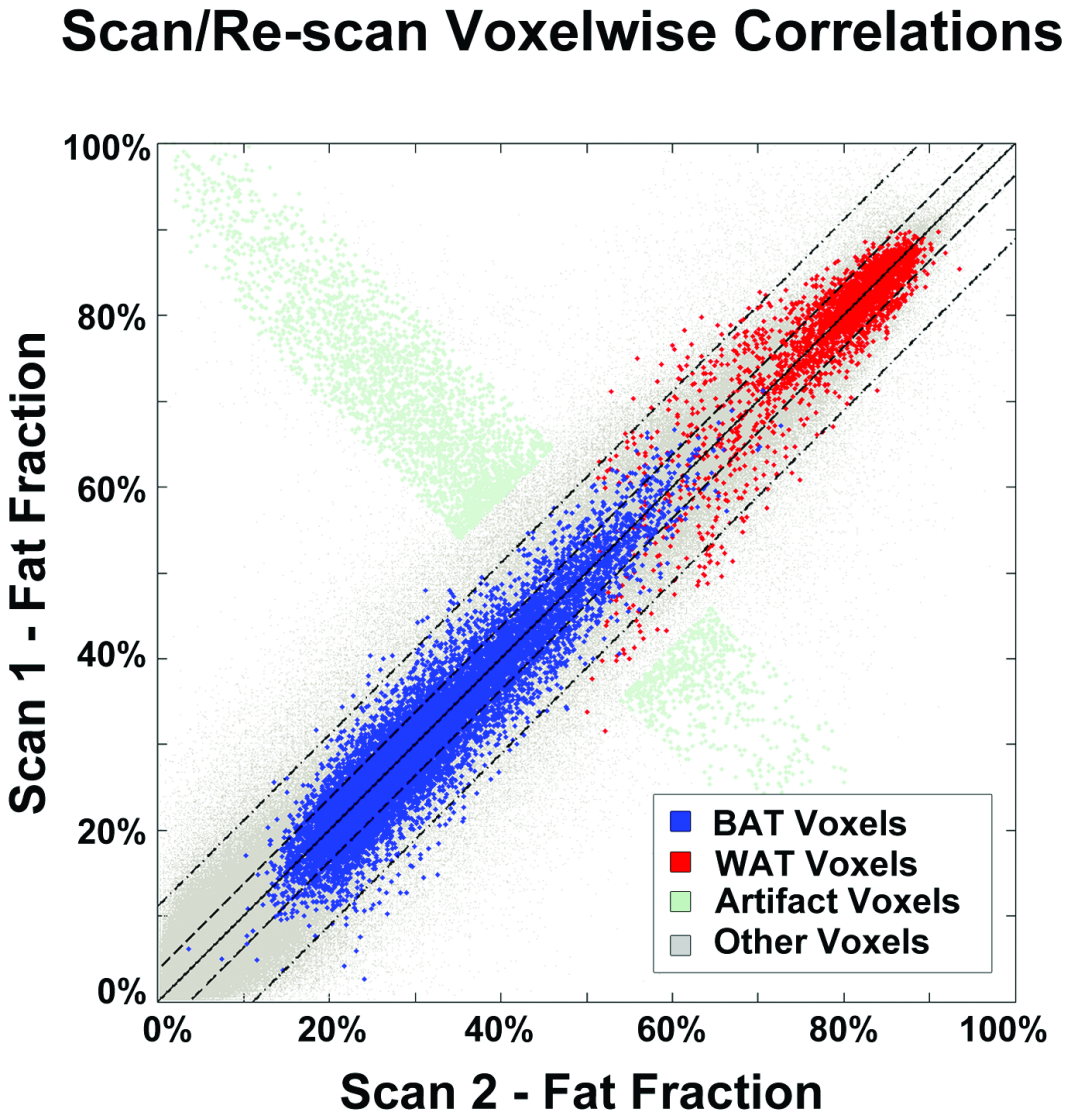
Voxelwise Repeatability. Voxel by voxel fat fraction correlation between scans. Strong voxelwise correlations between scans were observed in BAT segmentations (blue, R_BAT_ = 0.94), a WAT segmentation done in the nape of the neck (red, R_WAT_ = 0.88) and all other voxels (gray, R_ALL_ = 0.99). Artifact voxels are those identified in the hand.

The multi-echo water-fat MRI acquisition technique described was evaluated for test-retest reliability using a scan/re-scan approach examining voxelwise correlations between images of a single participant. Shim optimizations were run independently for each acquisition in order to reflect variation due to shim quality. The repeated acquisitions were separated only by the re-shim and default Siemens pre-scan preparations. The infant in-scanner position remained unchanged to maintain infant sleep state. There were no visually perceivable differences in position from the first to the second acquisition, although images were not co-registered to one another. Fat fraction quantitative and difference maps were made to assist the reader in visually assessing acquisition repeatability (Figure 5). After the image was masked for appreciable signal (as outlined above), voxelwise correlations were made and plotted for the union of all BAT segmentations, nuchal WAT and all remaining voxels (Figure 6).

**Figure 7:**
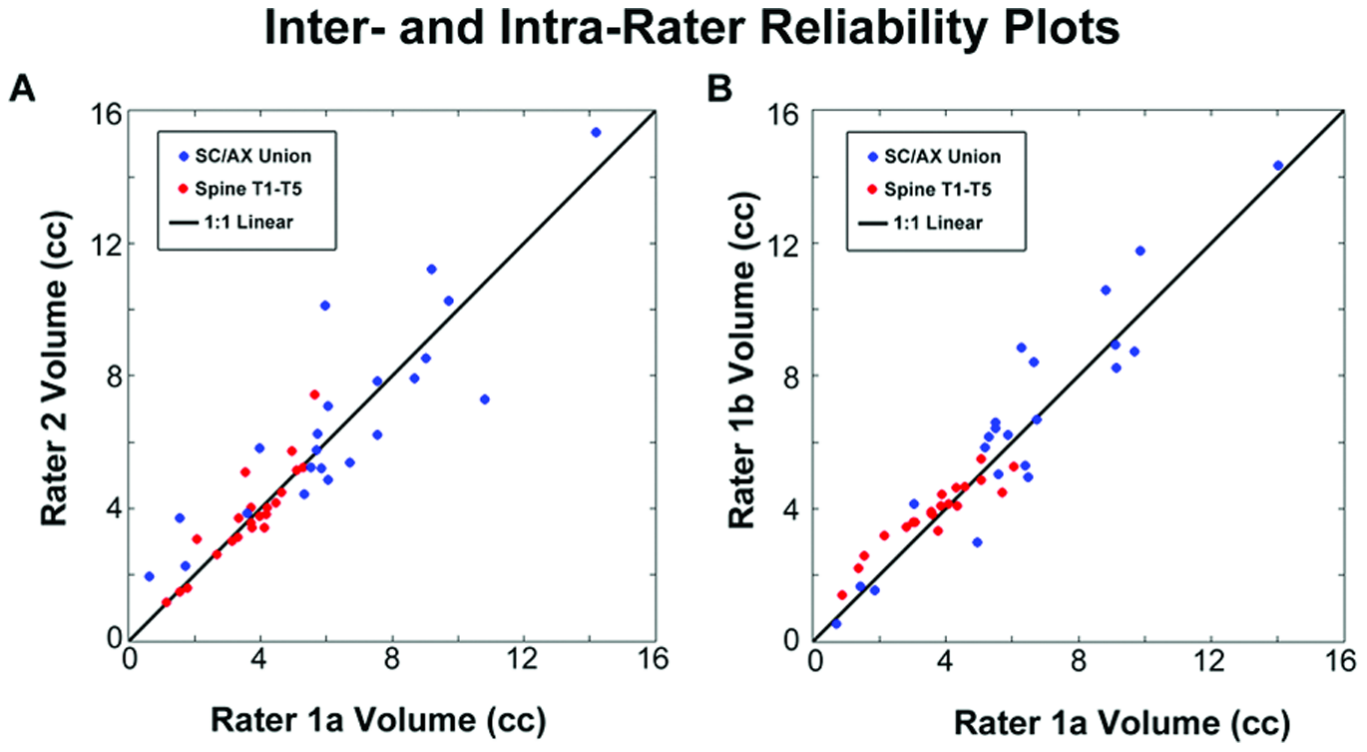
Segmentation Reliability. Plot of inter- and intra-rater depot volume reliability. (A) Brown Adipose Tissue (BAT) segmentations repeated on 22 subjects by two different raters: supraclavicular/axillary union ROIs (blue, R = 0.86) and spinal ROIs (red, R = 0.89). (B) BAT segmentations repeated on 22 subjects by a single rater separated by two weeks time (1a and 1b): supraclavicular/axillary ROIs (blue, R = 0.94) and spinal ROIs (red, R = 0.94).

Inter- and intra-rater reliability measures were used to characterize the repeatability of the novel methods used here in segmenting BAT deposits. Two raters (raters 1 and 2) segmented BAT deposits using the semi-automated techniques described above, with one rater (rater 1) repeating the segmentations of all 22 scans after two weeks (rating 1a, rating 1b) (Figure 7). Inter-rater reliability measure was then defined as the average intra-class correlation coefficient (ICC, the ratio of between-subject variance to total variance) between rating 1a vs. rating 2 and rating 1b vs. rating 2. The intra-rater reliability was taken as the ICC between rating 1a vs. rating 1b. Similar metrics referred to here as within-subject correlation coefficients (WSC, the ratio of within-subject or between-rater variance to total variance) and noise (N, the ratio of unaccounted for variance to total variance) are reported [24], [25].

## Results

### Rodent Ex Vivo Imaging

In the rats, mean excised BAT and WAT voxel FFs were significantly different (*p*_WAT-BAT_<10^−5^). *Ex vivo* iBAT_FF_ (43.7±7.8% SD) was 30% lower than perirenal WAT_FF_ (73.3±10.4%) (Figure 2). Excised WAT tissue was found to have a larger spread in FF values as well as a relatively asymmetric distribution (skewness = −0.85) as compared to that of BAT tissue (skewness = −0.27). The apparent gamma-like distribution of WAT is due to the limit on the maximum value observed.

### Rodent In Situ Imaging

In all 6 rodents, iBAT regions were qualitatively identifiable between the scapulae. Subcutaneous adipose tissue deposits were not accurately resolved due to the lower limit on spatial resolution for this protocol. The larger and more isotropic perirenal WAT deposits were easily identified. *In situ* relative to *ex vivo* FF differences were 0.8% and 5.4% in iBAT and WAT respectively (*p*_WAT-BAT_<10^−5^, iBAT_FF_ = 43.0±14.1%, WAT_FF_ = 67.9±15.6%). However, *in situ* compared to ex vivo measurement of iBAT FF showed a nearly two-fold larger standard deviation (SD_in situ_/SD_ex vivo_ = 1.81).

### Human Neonate Imaging

All 22 infants showed strong evidence for BAT deposits in spine, supraclavicular, and axillary regions (Figure 3). Spine, supraclavicular, axillary and union BAT region (Figure 4) fat fractions and depot volumes are reported in Table 1. Depot volume was more variable than FF in all regions, with the greatest depot volume variability found in supraclavicular BAT. Subcutaneous nuchal WAT had a mean FF of 67.7±4.6%. BAT and WAT FF means were significantly differentiated, pairwise, *in vivo* (Δ_WAT-BAT_ = 38%, *p*<10^−4^). In all four regions, FF was significantly positively correlated with depot volume (R_spine_ = 0.84, *p*_spine_<10^−3^; R_sc_ = 0.50, *p*_sc_<.05; R_axillary_ = 0.70, *p*_axillary_<10^−3^; R_union_ = 0.56, *p*_union_<.05).

**Table 1:** Summary of Brown Adipose Tissue Depot Volume and Fat Fraction.

	Volume (cc)		FF (%)	
	Mean+/−SD	Range	Mean+/−SD	Range
**Supraclavicular**	2.95+/−1.32	0.53–6.13	28.9+/−4.0	21.9–40.0
**Axillary**	3.76+/−2.00	0.52–8.72	30.3+/−3.3	24.9–35.3
**Union**	6.50+/−3.10	1.05–14.60	29.5+/−3.7	22.9–35.2
**Spine T1–T5**	3.65+/−1.40	1.02–6.70	32.2+/−3.2	25.4–38.1

Fat fraction defined as the ratio of fat signal to the sum of fat and water signal on a voxelwise basis.

### Acquisition Test-Retest Reliability

Fat fraction maps derived from two independent acquisitions of a single participant were visually indistinct from one another. The highest intensities found in the fat fraction difference map were limited to the exterior border interface of fat/air and a small region within the hand containing a phase swap artifact. The mean residuals between scan and re-scan were 2.7% in BAT, 2.6% in WAT and 2.8% across the entire image. Voxelwise fat fractions were strongly correlated with one another in all segmented BAT voxels (R_BAT_ = 0.94), subcutaneous nuchal WAT voxels (R_WAT_ = 0.88) and all remaining voxels (R_ALL_ = 0.99). The mean fat fraction difference between BAT and WAT was 18-fold greater than the mean residual between scans, suggesting a high level of BAT/WAT tissue differentiation repeatability from scan to scan.

### Inter and Intra-Rater Reliability

Inter- and intra-rater measurements of regional BAT depot volume and FF showed a high degree of reliability for all four ROIs examined in this study (Table 2). Spinal and supraclavicular/axillary union BAT depot volumes had the highest degree of between-rater reliability, followed by the supraclavicular region. Despite assessments being performed 2 weeks apart, intra-rater reliability was found to be slightly higher than inter-rater reliability. With the exception of axillary fat fraction, inter- and intra-rater performance of fat fraction characterization exceeded that of depot volume quantification. The supraclavicular/axillary union and bilateral spinal regions were seen to be the most robust to rater differences. The predominantly high intra-class correlations (Table 2) demonstrate the ability of the applied segmentation method to reliably measure BAT FF and depot volume in infants.

**Table 2:** Inter- and intra-rater reliability results.

	Depot Volume						Fat Fraction					
	Inter-Rater			Intra-Rater			Inter-Rater			Intra-Rater		
	ICC	WSC	N	ICC	WSC	N	ICC	WSC	N	ICC	WSC	N
**Supraclavicular**	0.61	0.11	0.28	0.74	0.04	0.22	0.83	0.00	0.18	0.89	0.00	0.11
**Axillary**	0.79	0.07	0.14	0.94	0.00	0.06	0.41	0.04	0.54	0.74	0.15	0.11
**Union**	0.86	0.00	0.14	0.93	0.00	0.07	0.91	0.00	0.09	0.93	0.02	0.06
**Spine**	0.87	0.00	0.13	0.89	0.01	0.10	0.96	0.01	0.04	0.97	0.00	0.03

Intraclass correlation coefficients (ICC) are given for segmentations performed between raters (inter-) and repeated within one rater (intra-). The union is the union of masks for supraclavicular and axillary ROIs. WSC (ratio of within-subject or between-rater variance to total variance) and N (ratio of unaccounted for variance to total variance) are given as complimentary measures.

## Discussion

This study examined the reliability of a non-invasive method for infant BAT imaging based on imaging in rodents and a relatively large cohort of neonates. Previous findings were replicated by demonstrating that multi-echo water-fat MRI-based BAT FF is significantly lower than WAT FF in both rodents (Δ_WAT-BAT_ 30%) and neonates (Δ_WAT-BAT_ 38%). Neonatal BAT deposit fat fraction and depot volume were quantified using MRI in supraclavicular regions, axillary regions, and bilaterally along the spine. The study findings show high BAT scan/re-scan and segmentation (fat fraction and BAT depot volume) reliability.

The BAT imaging protocol used in this study was validated in rodents by demonstrating that WAT and BAT tissues significantly differ in FF. Recent studies have established, albeit with limited data, that FF in BAT deposits are comparable between rodents and humans. The measured rodent BAT FF in our study (43%) is in tight concordance with previous observations [13]. To the best of our knowledge, only 3 published reports exist of human neonate BAT FF: the first was a *post mortem* infant with a measured fat fraction of 42% [16], the second was limited to two neonate subjects with fat fractions of 39% and 52% [18] , and the third had an infant age range from birth of up to six month with an average of 38.2% BAT fat fraction [19]. Closer inspection of infants of similar age in the third report suggests a range of FF from 20–40% (with an average of approximately 30%). These numbers overlap with the means and ranges observed in our report (BAT_FF,Range_ = 22–40%, BAT_FF_ = 30.2%). Finally, one study [11] reported a 10% increase in FF from the *in vivo* to *post mortem* state in rodents. Our findings support this observation, with supraclavicular BAT deposits for *in vivo* neonates having an average fat fraction of 29% compared to the post mortem fat fraction of 43% observed here in rats. The significant decrease in FF from the *in vivo* to the *ex vivo* state might be a physiological consequence of the perfusion that occurs in functioning BAT [26], contributing to the overall water content in BAT FF measurements.

Fat fraction as a relative measure is less susceptible to rater interpretation than volume measurements. BAT deposits are surrounded by a number of soft tissues, making their borders difficult to consistently define without a standardized protocol. Only 2 of the 4 previously published neonatal BAT MRI studies provide quantitative data on BAT depot volume. The first identified a unique iBAT deposit in 8 post-mortem infants with a mean (SD) of 3.6 mL (2.4). The second quantified a supraclavicular BAT volume of 17.4 ml in a single *post mortem* neonate. While this value is greater than the highest value observed here, the difference might easily be accounted for by a difference in age at scan (roughly 10 weeks on average). It should also be noted that current fat fraction methods fail to account for potential partial voluming of WAT interspersed into BAT depots, introducing an ambiguity to the true volume of BAT available for thermogenesis. Assuming uniform function and distribution of BAT, volume is important in the calculation of overall energy demand by the tissue. That means, more BAT volume equates to more available tissue for thermogenesis and consequently energy expenditure.

Prior literature has focused only on the supraclavicular/axillary regions of BAT in the human neonate. Our work measures other BAT deposition sites based on prior evidence from PET/CT and/or post mortem dissections. BAT deposition was consistently observed in three areas roughly corresponding to medial shoulder (supraclavicular), an area connecting the axilla to the inner shoulder (axillary), and bilaterally along the spine. The borders between the distribution in the axillary regions and those within supraclavicular regions were difficult to differentiate in some subjects. The spinal deposits along the transverse and articular processes in the spinal column were often accompanied by deposition around the vertebral body and the spinous process. For consistency, only the deposits along the transverse and articular processes were segmented in this study and limited to vertebrae T1–T5. The BAT regions described here were sometimes accompanied by a bilateral distribution extending up the neck, but were not segmented due to their inconsistent presence.

All neonate BAT regions identified in this study are concordant with regions identified in adult PET studies [1]–[3], [27]. Recent documentation of *in vivo* BAT in infants [19] more fully characterizes the fat fraction in the supraclavicular fossa and describes the low fat fraction region extending into the nape of the arm. A clinical report more fully describes the axillary deposit seen in the neonate.^13^ In early reports the spinal region is discussed as the predominant site of heat production in the neonate [28], [29]. All of these regions are visible in PET/CT studies, with more pronounced signs of metabolic activity in the supraclavicular regions. It is not known to what extent the regions examined in these studies overlap, and what influence *post mortem* effects may have had on previous attempts at detecting BAT throughout the torso.

An assessment of various aspects of reliability was performed in order to validate the consistency of the methodology used here. Spinal adipose deposits present with clearly delineated borders resulting in a high degree of measurement reliability within and between raters. Supraclavicular regions posed a more significant challenge for segmentation routines due to the presence of other soft tissues and a relatively ambiguous border between the supraclavicular and axillary areas. Each of these regions on their own showed a high degree of reliability within a rater, but was found to be more rater dependent when compared to the union of supraclavicular and axillary regions. In order to minimize rater bias it is our recommendation based on these observations that in the absence of clear definable anatomical boundaries the combined region including the axillary be used in association studies.

As is the case for any newly developing field, there is more to be done in the area of neonatal BAT imaging. We have demonstrated reliable methods that are readily available to researchers. More sophisticated, but less clinically accessible, methods of quantifying the proton density fat fraction are capable of accurate quantification of water and fat by using multiple echoes (3+) to model multiple fat peaks, T1/T2/T2* relaxation and other potential confounds [30]. Despite the increased accuracy extra echoes affords in water-fat separation, multiple methods have demonstrated the capability of dual-echo methods in discriminating BAT from WAT in humans [17], [31]. Because PET/CT presents unnecessary risks to healthy pediatric populations, water-fat MRI is currently the most viable solution for *in vivo* quantification of BAT composition.

MRI methods, however, have yet to provide a direct measurement of BAT activity and thermogenesis in neonates as do PET/CT and thermography, respectively. Furthermore, multi-echo water-fat MRI does not differentiate between active and inactive BAT, a potential confound when considering the biological importance of BAT activity. Current MRI modalities capable of this may include imaging the perfusion associated with BAT activation using Arterial Spin Labeling or applying methods established for Blood-Oxygen-Level-Dependent changes in rodents [32] under norepinephrine administration or humans under cold exposure [33]. Two major obstacles prevent temperature challenges in assessing BAT function in neonates: 1) safely and reliably exposing neonates to standardized cold conditions, and 2) detecting small *in vivo* signal changes induced by BAT activation brought on by cold condition administration. Diffusion Tensor Imaging may also present an interesting avenue for descriptive work due to its known ability to demonstrate diffusion in adipose tissue [34] as well as the ability to co-locate the nerve endings needed for norepinephrine innervation of BAT [35]. Finally, hyperpolarized ^13^C imaging has recently been used to monitor BAT activation in rodents under norepinephrine injection by measuring the metabolic conversion of pre-polarized [1-^13^C] pyruvate [36] and shows great promise in BAT activity detection.

Our study imaged, identified, quantified and examined the reliability of BAT composition in neonates. Currently, little is known in humans about the developmental determinants of BAT and the changes in BAT mass during the first years of life. Given the suggestion that early reductions in BAT deposition may continue throughout life [37] and may be related to the onset of obesity, localizing and quantifying BAT depot volume and fat fraction in newborns and infants may help us arrive at a better understanding of the underlying early developmental risk factors for obesity and metabolic dysfunction. The application of these methods holds promise towards this goal and therefore has important implications for health and disease risk over the individual lifespan.
