# Correction: Brown Adipose Tissue Quantification in Human Neonates Using Water-Fat Separated MRI

**DOI:** 10.1371/annotation/f1b2fa68-726c-4f30-b061-d05c8a281eb8

**Published:** 2014-01-09

**Authors:** Jerod M. Rasmussen, Sonja Entringer, Annie Nguyen, Theo G. M. van Erp, Joshua Burns, Ana Guijarro, Fariba Oveisi, James M. Swanson, Daniele Piomelli, Pathik D. Wadhwa, Claudia Buss, Steven G. Potkin

Joshua Burns is incorrectly omitted from the author byline. He should be listed as the fifth author and affiliated with institution number 1. The contributions of this author are as follows: Analyzed the data.

The correct Citation is: Rasmussen JM, Entringer S, Nguyen A, van Erp TGM, Burns J, et al. (2013) Brown Adipose Tissue Quantification in Human Neonates Using Water-Fat Separated MRI. PLoS ONE 8(10): e77907. doi:10.1371/journal.pone.0077907

